# Efficacy and Safety of Tranexamic Acid in Sleeve Gastrectomy: A Double-Blind Randomized Controlled Trial

**DOI:** 10.1007/s11695-026-08496-6

**Published:** 2026-02-16

**Authors:** Mohammed Elshwadfy Nageeb, George Abdelfady Nashed, Mohamad Alaa Eldin Atef Elzayat, Mohamed Nasr Shazly

**Affiliations:** Department of General Durgery, Kasr Alainy Faculty of Medicine, Cairo University, Giza, Egypt

**Keywords:** Tranexamic acid, Sleeve gastrectomy, Bleeding, Bariatric surgery

## Abstract

**Background:**

Intraoperative bleeding during sleeve gastrectomy (SG) can complicate dissection and increase transfusion risk. Tranexamic acid (TXA) is an antifibrinolytic agent with proven efficacy in reducing surgical blood loss, but its role in metabolic and bariatric surgery (MBS) remains uncertain.

**Methods:**

A single-center, randomized, double-blinded trial including 132 patients undergoing laparoscopic SG between July 2024 and July 2025 was conducted. Participants were randomized to receive 1 g intravenous TXA before incision or placebo. The primary outcome wasintroperative estimated blood loss (EBL).Perioperative estimated blood loss and Hemoglobin decline (ΔHb) served as key secondary confirmation. Secondary outcomes included intraoperative hemostatic measures, perioperative complications, and length of stay. Analyses were performed using intention-to-treat.

**Results:**

The mean intraoperative estimated blood loss (EBL) was significantly lower in the TXA group compared with controls (118.6 ± 42.7 mL vs. 164.3 ± 51.2 mL, *p* < 0.001). Meanperioperative EBL was significantly lower in the TXA group compared with controls (270.4 ± 110.5 mL vs. 392.2 ± 136.6 mL, *p* < 0.001). Postoperative hemoglobin decline was also smaller with TXA (0.5 [0.4–0.8] g/dL vs. 0.9 [0.6–1.1] g/dL, *p* < 0.001). Patients receiving TXA required fewer intraoperative hemostatic clips and had fewer bleeding points. Operative time and length of stay were similar. No thromboembolic events, transfusions, or deaths occurred within 30 days. Complication rates did not differ between groups.

**Conclusions:**

A single preoperative dose of TXA reduced intraoperative blood loss and early hemoglobin decline in SG without evidence of increased short-term complications. Confirmation in larger multicenter cohorts is warranted before considering broader adoption in MBS practice.

## Key Points

• Tranexamic acid (TXA) significantly reduced intraoperative blood loss during sleeve gastrectomy (SG).

• TXA use was associated with a smaller decline in hemoglobin on postoperative day 1.

• No thromboembolic events, transfusions, or deaths were observed within 30 days.

• Safety outcomes and complication rates were similar between TXA and control groups.

## Introduction

Metabolic and bariatric surgery (MBS) remains the most effective treatment for severe obesity, yet perioperative bleeding continues to be a clinically significant concern. Sleeve gastrectomy (SG), the most frequently performed MBS procedure worldwide [1, 2], is associated with a postoperative bleeding rate of 2–4%, often requiring transfusion, reoperation, or prolonged hospitalization [3] despite ongoing refinements in safety measures [4, 5]. Conventional measures such as staple-line reinforcement, alternative staple configurations, and energy devices have shown variable effectiveness, and pharmacological agents such as tranexamic acid (TXA) are being investigated as complementary strategies [3].

TXA, a synthetic antifibrinolytic agent, has become a cornerstone of blood-conservation strategies across numerous elective surgical specialties—including orthopedic joint arthroplasty, cardiac, spinal, craniofacial, and obstetric surgery—where it reliably reduces perioperative blood loss and transfusion requirements without compromising safety [3, 6, 7]. Large multicenter randomized trials and meta-analyses encompassing tens of thousands of patients have demonstrated that TXA does **not** increase venous thromboembolism (VTE) or other thrombotic complications in these settings [8, 9]. Despite this reassuring safety profile, TXA remains under-utilized in metabolic and bariatric surgery because of theoretical concerns about the already elevated baseline VTE risk in obese patients—an uncertainty that underscores the need for procedure-specific, evidence-based evaluation.

In MBS, patients present a paradoxical risk profile: bleeding during staple-line dissection versus an elevated baseline risk of venous thromboembolism (VTE) [3, 6]. This has historically limited TXA adoption, but recent evidence has challenged this caution.

Randomized trials in SG show that intravenous TXA significantly reduces intraoperative bleeding, hemoglobin decline, and staple-line interventions without increasing complications [10–12]. A prospective controlled study also demonstrated reduced reoperation and length of stay with TXA, with no thromboembolic events [13]. Meta-analyses pooling over 1,100 patients confirm that TXA approximately halves the odds of postoperative bleeding, shortens operative duration and hospitalization, and does not increase VTE or mortality [6].

Although a trend toward lower transfusion requirements was noted with TXA, this did not reach statistical significance, likely owing to the low overall frequency of transfusions in MBS [7]. Crucially, no increase in thromboembolic events has been reported across high-quality reviews [7], addressing the major safety concern. Nonetheless, heterogeneity in dosing, timing, and administration persists, and most trials remain single center with modest sample sizes. Further multicenter studies are needed to define the optimal regimen and confirm safety across diverse MBS populations. Against this background, we conducted a randomized, double-blinded trial to evaluate the efficacy and safety of a single preoperative intravenous dose of TXA during SG.

## Methods

### Study Design and Setting

This was a prospective, randomized, double-blinded controlled trial conducted at universityteaching Hospitals, a high-volume tertiary referral center for MBS. The study period extended from July 2024 and July 2025. The protocol was reviewed and approved by the institutional review ethics committee boardbefore study initiation, and all procedures were performed in accordance with the Declaration of Helsinki and the CONSORT 2010 statement [14]. Written informed consent was obtained from all participants before enrollment.

### Participants

Patients aged 18–60 years were eligible if they had a body mass index (BMI) ≥ 35 kg/m², or ≥ 30 kg/m² with at least one obesity-related disease (type 2 diabetes, hypertension, or obstructive sleep apnea). All participants were considered suitable candidates for primary sleeve gastrectomy (SG) accordingto the 2022 ASMBS/IFSO criteria [15]. Patients were excluded if they had a known coagulopathy or platelet disorder, a history of venous thromboembolism, myocardial infarction, or cerebrovascular accident, or if they on anticoagulant or antiplatelet therapy within ten days before surgery. Additional exclusion criteria included prior bariatric or major upper abdominal surgery, allergy or hypersensitivity to tranexamic acid, chronic kidney disease (stage 4–5), significant hepatic impairment, pregnancy or lactation.

### Randomization and Blinding

Participants were randomized in a 1:1 ratio to receive tranexamic acid or placebo using a computer-generated block randomization sequence (block size = 4) prepared by an independent pharmacist. Allocation concealment was maintained with sequentially numbered, opaque, sealed envelopes. The pharmacist also prepared indistinguishable infusion bags containing either tranexamic acid (1 g diluted in 100 mL of 0.9% saline) or placebo (100 mL of 0.9% saline). The infusion was administered intravenously over 10–15 min immediately after induction of anesthesia and before skin incision, at a maximum rate of 100 mg per minute. Blinding was maintained for patients, anesthesiologists, surgeons, scrub nurses, and all postoperative assessors. All analyses were performed according to the intention-to-treat principle.

### Anesthesia Protocol

Anesthesia was standardized according to ASA and ERAS recommendations for bariatric surgery. Induction was achieved with propofol (2 mg/kg lean body weight), fentanyl (2 µg/kg), and atracurium (0.5 mg/kg). Maintenance was with isoflurane (MAC 1.2–1.5%) in a 50% O₂/air mixture.

Multimodal analgesia included paracetamol 1 g IV, ketorolac 30 mg IV, dexamethasone 8 mg IV, and magnesium sulfate 30 mg/kg IV (used as an analgesic adjunct to reduce anesthetic requirements and postoperative pain). Antiemetic prophylaxis consisted of ondansetron 4 mg IV and dexamethasone. Ceftriaxone 1 g IV was administered before induction per institutional infection control policy.

All patients received intraoperative crystalloid fluids at a standardized rate of 5 mL/kg/hour, calculated based on ideal body weight. Then adjusted fluid rate according to intraoperative monitoring.

### Tranexamic Acid Administration

Patients in the intervention arm received 1 g of tranexamic acid diluted in 100 mL of saline, infused intravenously over 10–15 min immediately after induction of anesthesia and before skin incision, at a maximum rate of 100 mg per minute. Controls received an identical 100 mL saline infusion. Infusion bags were prepared by an independent pharmacist to ensure allocation concealment and blinding of patients, anesthetists, surgeons, and assessors.

### Surgical Protocol

All sleeve gastrectomies were performed laparoscopically by the same bariatric team using a standardized five-port approach. Gastric mobilization extended from 4 cm proximal to the pylorus to the angle of His, with vascular control achieved using the LigaSure™ vessel sealing system (Medtronic). Resection was performed over a 36-French bougie. All procedures employed standard Covidien GIA™ staplers without buttressing, and omental or perigastric bleeding points were controlled using titanium hemostatic clips, and the number of hemostatic clips used was recorded as a trial endpoint. Surgical drains were not routinely placed.

### Postoperative Management

Patients were monitored in the surgical ward with vital signs recorded every two hours. Analgesia consisted of scheduled intravenous paracetamol and ketorolac, with rescue morphine if pain scores were ≥ 4. Supportive therapy included IV isotonic crystalloid at 2 mL/kg/hour (ideal body weight) until the patient tolerates oral intake ≥ 1 L, then discontinue IVmaintenance, pantoprazole, and antiemetics. Enoxaparin 40 mg was started 12 h postoperatively if hemostasis was secured and continued for 14 days, together with mechanical prophylaxis until mobilization. Patients were reviewed weekly in the first postoperative month and monthly thereafter for one year.

### Outcomes

The primary endpoint was intraoperative estimated blood loss (EBL), calculated from suction output plus gauze weight (EBL = (Suction volume – Irrigation fluid volume) + (Weight of used gauze – Weight of dry gauze)). Perioperative estimated blood loss and Hemoglobin decline (ΔHb) served as a key secondary confirmationHemoglobin decline (ΔHb), defined as preoperative hemoglobin minus postoperative day 1 hemoglobin (g/dL).Perioperative estimated blood loss (which included total blood loss (Vloss), intraoperative blood loss, and occult blood loss) was calculated using the hemoglobin balance method.

### Blood Volume Loss (Vloss total)

Vloss total = 1000 × Hbloss total/Hbi.

Hemoglobin Loss (Hbloss).

Hbloss total = BV × (Hbi − Hbe) × 0.001 + Hbt.

BV (L): blood volume - Hbloss total (g): volume of Hbloss-Hbi (g/L): preoperative Hb value- Hbe (g/L): postoperativeHb value - Hbt (g): total blood transfusion volume.

The estimated blood volume (BV) is often calculated using Nadler’s formula: (Men: 70 mL/kg × body weight (kg) - Women: 65 mL/kg × body weight (kg) Men: 75 mL/kg).The factor 0.001 is to convert from mL·g/L to grams of hemoglobin lost.

Secondary endpoints included the number of bleeding points requiring hemostatic clip application, operative time, postoperative white blood cell and platelet counts on day 1, and clinical complications such as melena, vomiting or transfusion. Safety endpoints included the occurrence of symptomatic venous thromboembolism within 30 days, diagnosed clinically and confirmed by duplex ultrasound or computed tomography, and 30-day mortality. Hospital length of stay (hours) and reoperation for bleeding were recorded as exploratory outcomes. These outcomes were chosen to align with prior randomized trials evaluating tranexamic acid in sleeve gastrectomy [10–12].

### Sample Size Calculation

Sample size was estimated using G*Power (version 3.1) for a two-tailed independent samples t test with the primary endpoint of estimated blood loss (EBL). Assuming a medium effect size (Cohen’s d = 0.5), α = 0.05, and 80% power, the required sample was 128 participants (64 per group). This effect size was selected based on prior randomized trials in sleeve gastrectomy reporting mean EBL differences of 100–150 mL between TXA and control [10–12]. To account for attrition, we planned to recruit 154 patients. Ultimately, 132 patients completed follow-up and were analyzed (66 per group).

### Statistical Analysis

Data analyses were performed using IBM SPSS Statistics for Windows, version 25.0 (IBM Corp., Armonk, NY, USA). The Shapiro–Wilk test was applied to assess normality of continuous variables. Normally distributed data were reported as mean ± standard deviation (SD) and compared between groups using the independent-samples t test. Non-normally distributed variables were reported as median (interquartile range, IQR) and compared using the Mann–Whitney U test. Categorical data were summarized as frequencies and percentages and compared using Fisher’s exact test.

The primary analysis followed the intention-to-treat principle. Multivariable linear regression was conducted to identify independent predictors of estimated blood loss (EBL) and postoperative hemoglobin decline (ΔHb). Candidate predictors included intervention group (TXA vs. control), BMI, operative time, age, sex, diabetes, and hypertension. Group allocation was coded with the control group as the reference category, and results were presented as adjusted mean differences with 95% confidence intervals (CI). All statistical tests were two-sided, and *p* < 0.05 was considered statistically significant.

## Results

### Baseline Characteristics

**Fig. 1:**
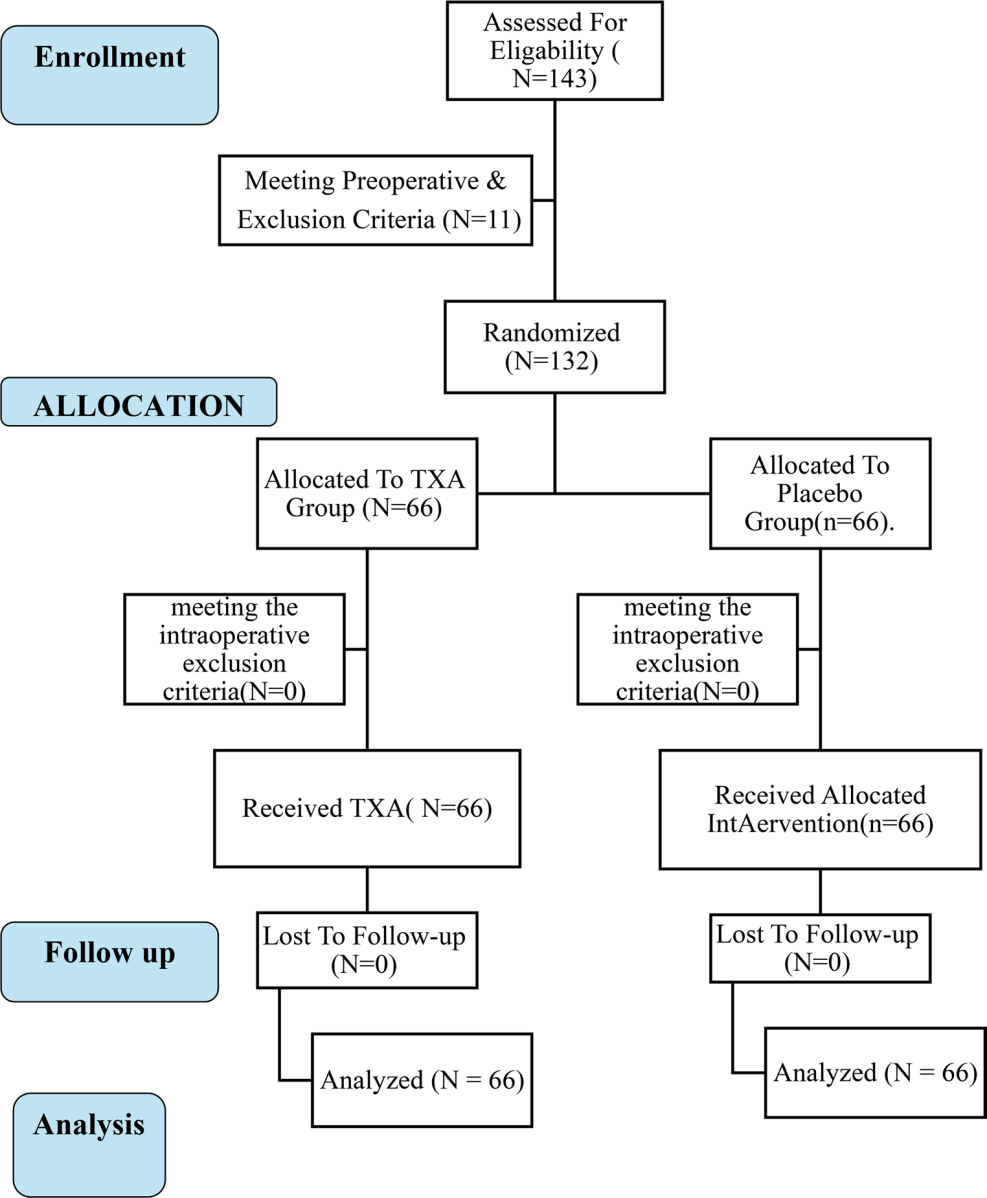
The Consort Flow Diagram

A total of 132 patients were randomized and completed the trial, with 66 assigned to the TXA group and 66 to the control group (Fig. 1). The median age was 43 (IQR 33.75–51) years in the TXA group and 43 (IQR 35–50.25) years in the control group (*p* = 0.75) (Table 1). Females comprised 74.2% of the TXA group and 63.3% of controls (*p* = 0.8) (Table 1). The preoperative weight was 125.5 (118–136.5) kg versus 125 (116.7–136) kg (*p* = 0.69), with corresponding BMIs of 45.17 ± 5.08 and 45.44 ± 5.28 kg/m² (*p* = 0.76) (Table 1).

**Table 1:** Baseline demographic and intraoperative characteristics

	TXA group	Control group	*P*-value
	(*n* = 66)	(*n* = 66)	
Age, year ^a^	43 (33.75–51)	43 (35–50.25)	0.755
Sex Female Male	49 (74.2) 17 (25.8)	51 (63.3) 15 (22.7)	0.839
Weight ^a^	125.5 (118–136.5)	125 (116.7–136)	0.692
BMI ^b^	45.17 ± 5.08	45.44 ± 5.28	0.763
Hypertension	11 (16.7)	10 (15.2)	1.000
Diabetes Mellitus	11 (16.7)	12 (18.2)	1.000
Other co-morbidities	11 (16.7)	10 (15.2)	1.000
Previous Surgeries	0 (0)	0 (0)	-
Type of surgery Sleeve	66 (100)	66 (100)	-
Operative time, min^a^	82.5 (73.75–95)	90 (75–100)	0.220
N. of Staples used ^a^	6 (5–6)	6 (5–6)	0.858
HemostaticClips ^a^	4 (3–5)	5 (4–5)	0.002
N. of bleeding points ^a^	4 (3–5)	5 (4–5)	< 0.001
estimated blood loss (EBL) in ml ^b^	118.6 ± 42.7	164.3 ± 51.2	< 0.001
Intraoperative complications	0 (0)	0 (0)	-

All values presented in Number (percentage) unless indicated otherwise.

^a^ median (Q1-Q3)

^b^ mean ± SD

Preoperative hemoglobin was 13 (13–13.75)g/dL in the TXA group compared with 13 (13–13.5) g/dL in controls (*p* = 0.93) (Table 2). Estimated blood volume was similar (Table 2). Platelet counts were 218 ± 59 × 10³/µL and 216 ± 58 × 10³/µL (*p* = 0.74), while white blood cell counts were 6 (5–9)× 10³/µL and 7 (5–9)× 10³/µL (*p* = 0.43) (Table 2).

**Table 2:** Preoperative hematologic and coagulation profiles

	TXA group	Control group	Test statistic	*P*-value
	(*n* = 66)	(*n* = 66)		
Hb level(mg/dl)	13 (13–13.75)	13 (13–13.5)	U = 2159	0.926
Blood volume(L)	6.1 (5.65–6.36)	6.1 (5.56–6.31)	U = 1976	0.358
Platelets ^a^(10^3)^	218.48 ± 59.79	216.44 ± 58.82	t = 0.198	0.843
WBCs	6 (5–9)	7 (5–9)	U = 2351	0.425
PT(seconds)	13 (12–14)	13 (12–14)	U = 2396	0.292
INR	0.9 (0.8–1)	0.9 (0.8–1)	U = 2319	0.501

All values presented in median (Q1-Q3) unless indicated otherwise.

^a^ mean ± SD

Multivariate regression confirmed that none of the baseline variables significantly predicted estimated blood loss or postoperative hemoglobin drop. Specifically, age (β = 0.12, 95% CI − 0.08 to 0.32, *p* = 0.24), sex (β = − 15.3, 95% CI − 43.8 to 13.1, *p* = 0.29), BMI (β = 1.1, 95% CI − 1.6 to 3.9, *p* = 0.42), baseline hemoglobin (β = − 2.4, 95% CI − 7.3 to 2.6, *p* = 0.34), platelet count (β = 0.03, 95% CI − 0.02 to 0.07, *p* = 0.25), and estimated blood volume (β = − 0.004, 95% CI − 0.01 to 0.003, *p* = 0.30) were not independently associated with blood loss (Table 3). Similarly, none of these covariates predicted postoperative hemoglobin decline. These findings reinforce that randomization achieved well-balanced groups without baseline confounding.

**Table 3:** 30-day postoperative complications and Follow-up

	TXA group	Control group	*P*-value
	(*n* = 66)	(*n* = 66)	
Fever ^a^	4 (6.1)	5 (7.6)	1.000
Vomiting ^a^	7 (10.6)	8 (12.1)	1.000
Melena ^a^	1 (1.5)	2 (3)	0.780
DVT ^a^	0 (0)	0 (0)	-
Mortality ^a^	0 (0)	0 (0)	-
Hb variation	0.5 (0.4–0.8)	0.9 (0.63–1.1)	< 0.001
Perioperative EBL ^b^	270.38 ± 110.52	392.15 ± 136.59	< 0.001
Follow up periods, months	24 (24–30)	24 (24–30)	0.941
Hb level (mg/dl)	12.5 (12.1–13.2)	12.3 (11.8–12.6)	0.057
Platelets (10 ^3^)^b^	237.91 ± 62.26	234.67 ± 61.03	0.763
WBCs	9.4 (8.6 - 11.2)	10.3 (9.4–11.9)	0.029
PT	13.5 (12.5–14.4)	13.5 (12.5–14.4)	0.409
INR	1 (0.9–1.1)	1 (0.9–1.1)	0.516
Allogenic transfusion ^a^	0 (0)	0 (0)	-
Hospital stays in hours	24 (24–30)	28 (24–36)	0.226

All values presented in median (Q1-Q3) unless indicated otherwise.

^a^ number (percentage)

^b^ mean ± SD ^a^ number (percentage)

### Intraoperative Outcomes

The mean intraoperative estimated blood loss (EBL) was significantly lower in the TXA group compared with controls (118.6 ± 42.7 mL vs. 164.3 ± 51.2 mL, *p* < 0.001) (Table 1). Operative time was similar between groups (82.5 (73.75–95) minutes vs. 90 (75–100) minutes, *p* = 0.220) (Table 1). The number of bleeding points requiring hemostatic clip application was also reduced in the TXA group 4 (3–5) vs. 5 (4–5), p = 0.002) (Table 1).

In multivariate linear regression, assignment to the TXA group remained a strong independent predictor of lower blood loss (β = − 45.7, 95% CI − 63.2 to − 28.2, *p* < 0.001) after adjusting for age, sex, BMI, baseline hemoglobin, diabetes, hypertension, and operative time (Table 3). None of the covariates, including operative time (β = 0.8, 95% CI − 0.3 to 1.9, *p* = 0.15) or BMI (β = 0.9, 95% CI − 1.1 to 2.8, *p* = 0.38), were significantly associated with EBL. TXA use also independently predicted fewer bleeding points requiring hemostatic clipping (β = − 1.2, 95% CI − 1.7 to − 0.7, *p* < 0.001), confirming its intraoperative hemostatic benefit independent of patient- or surgery-related factors.

### Postoperative Hematologic Outcomes

Postoperative hemoglobin decline was also smaller with TXA (0.5 [0.4–0.8] g/dL vs. 0.9 [0.6–1.1] g/dL, *p* < 0.001). (Table 4). In multivariate regression, TXA administration remained a strong independent predictor of reduced hemoglobin loss (β = − 0.56, 95% CI − 0.78 to − 0.34, *p* < 0.001), after adjustment for age, sex, BMI, baseline hemoglobin, diabetes, hypertension, and operative time. Operative time (β = 0.02, 95% CI 0.01–0.03, *p* < 0.001) and baseline hemoglobin (β = 0.14, 95% CI 0.04–0.24, *p* = 0.005) were also independently associated with hemoglobin drop, whereas none of the other covariates were significant (Table 1)(Table 3).

**Table 4:** Multivariate linear regression analysis for predictors of estimated blood loss and hemoglobin loss

Predictors		B (Unstandardized)	95% CI for B	*P*-value
Estimated Blood Loss (EBL)	TXA vs. Control group	−117.617	−160.97- −74.27	< 0.001
	BMI	−1.109	−5.66–3.44	0.631
	Operative time (min)	1.263	−0.36–2.89	0.126
	Age (years)	−1.313	−3.68–1.05	0.274
	Gender	−11.337	−63.41–40.73	0.667
	DM	25.093	−34.04–84.23	0.403
	HTN	−10.890	−72.25–50.46	0.726
Hemoglobin Loss	TXA vs. Control group	0.016	0.01–0.22	< 0.001
	BMI	< 0.001	0–0.001	0.351
	Operative time (min)	< 0.001	0–0.001	0.122
	Age (years)	< 0.001	−0.001–0	0.115
	Gender	< 0.001	−0.007–0.008	0.895
	DM	0.005	−0.003–0.013	0.236
	HTN	−0.001	−0.009–0.008	0.879

Postoperative WBC was significantly lower in the TXA group compared with controls (median 9.4 [IQR 8.6–11.2] vs. 10.3 [IQR 9.4–11.9], *p* = 0.029) (Table 4). Platelet counts were comparable between groups (237.9 ± 62.3 × 10³/µL vs. 234.7 ± 61.0 × 10³/µL, *p* = 0.76) (Table 4). Regression analysis confirmed that TXA administration had no independent association with platelet count, whereas the association with lower WBC approached significance but did not remain robust after adjustment (Table 4).

### Clinical Outcomes and Safety

No patients in either group required intraoperative blood transfusion (Table 3). Melena was reported in 2 patients (3.0%) in the control group compared with 1 patient (1.5%) in the TXA group (*p* = 0.56) (Table 4). Postoperative vomiting occurred with similar frequency in both groups (10.6% vs. 12.1%, *p* = 0.78) (Table 4), and no patients in either arm experienced hematemesis or blood-streaked vomitus.

Symptomatic venous thromboembolism within 30 days was not observed in either group, or there were no deaths during the 30-day follow-up (Table 4).

In multivariate regression, none of the clinical or safety outcomes, including melena, or vomiting, were independently associated with TXA use after adjusting for age, sex, BMI, operative time, and baseline hemoglobin (Table 3).

## Discussion

This randomized controlled trial demonstrates that tranexamic acid (TXA) significantly reduces intraoperative blood loss and early hemoglobin decline in sleeve gastrectomy without increasing complications. Patients receiving TXA also required fewer hemostatic clips, reflecting improved intraoperative hemostasis.

The antifibrinolytic effect of TXA, blocking plasminogen activation and stabilizing fibrin clots, is well established [8, 16].Our findings extend prior evidence from orthopedic, cardiac, and obstetric surgery to MBS, where the balance between bleeding risk and venous thromboembolism (VTE) risk has been a concern.

### Reduction in Estimated Blood Loss (EBL) and Hemoglobin Loss

The key finding of this trial was the significant reduction in estimated blood loss and postoperative hemoglobin decline with tranexamic acid (TXA). Mean EBL and hemoglobin loss were both significantly lower in the TXA group compared with controls (*p* < 0.001), confirming its hemostatic benefit.

Reduced bleeding not only decreases the risk of transfusion but also improves surgical field visibility and may shorten operative time. Although transfusion was not required in either group, the attenuation of hemoglobin loss underscores TXA’s systemic effect.

These results are consistent with prior randomized trials and meta-analyses demonstrating reductions in EBL and hemoglobin loss with TXA in SG [3].

### Intraoperative Hemostasis Metrics

In addition to the quantitative reduction in blood loss, our trial demonstrated improved intraoperative hemostasis with tranexamic acid (TXA). Patients in the TXA group required fewer hemostatic clips and had fewer bleeding points compared with controls, reflecting a more stable surgical field. These findings suggest that TXA reduces the burden of minor oozing, which not only facilitates hemostasis but also enhances surgical visibility and may contribute to operative efficiency [12].

The reduced reliance on hemostatic clips may also carry practical implications, including decreased operative cost and fewer foreign materials left in situ [12]. Importantly, while the absolute differences inhemostatic clip usage appear modest, they indicate a consistent trend that aligns with TXA’s antifibrinolytic mechanism, which stabilizes clot formation at the microvascular level.

Similar improvements in surgical field quality have been reported in orthopedic and cardiac procedures, where TXA reduces diffuse bleeding and the need for adjunctive hemostatic measures [9, 12, 17]. Our findings extend this observation to SG, suggesting that TXA not only lowers blood loss but also enhances the overall quality of intraoperative hemostasis.

### Postoperative Outcomes and Novel Observations

Although the difference in postoperative hemoglobin did not reach statistical significance (*p* = 0.057), the trend toward higher levels in the TXA group (12.5 g/dL vs. 12.3 g/dL) may still carry clinical relevance. Even modest preservation of hemoglobin can support faster recovery, reduced fatigue, and improved well-being during the early postoperative course. This near-significant signal suggests that TXA’s hematological benefit may be more evident in larger cohorts or in patients at higher bleeding risk.

Interestingly, patients receiving tranexamic acid exhibited slightly lower postoperative white blood cell counts compared with controls. While this difference reached statistical significance, (median 9.4 vs. 10.3; *p* = 0.029), it should be interpreted as an exploratory, hypothesis-generating observation rather than evidence of a direct anti-inflammatory effect. Reduced WBC counts may reflect attenuated surgical stress or lesser tissue trauma due to improved hemostasis, rather than a pharmacological anti-inflammatory property of TXA. Experimental studies have suggested that TXA may influence inflammatory mediators and leukocyte activation; however, these findings remain preliminary and inconsistent. Larger mechanistic trials incorporating cytokine profiling are required to determine whether TXA exerts a genuine immunomodulatory effect or whether this association is secondary to improved intraoperative hemostasis [18].

While exploratory, this observation highlights a potential systemic effect of TXA beyond its antifibrinolytic role. If confirmed in future studies, TXA may offer dual advantages in metabolic and bariatric surgery: minimizing bleeding while simultaneously blunting the inflammatory response. This warrants further investigation, as it may expand the clinical rationale for TXA use in surgical practice.

#### Safety Profile and Complications

TXA demonstrated an excellent safety profile: no cases of deep vein thrombosis (DVT) or mortality were observed in either group. Postoperative complications such as fever, vomiting, or bleeding were rare and occurred at comparable rates, with no statistically significant differences.

These findings align with large-scale studies and meta-analyses across multiple surgical fields, including MBS, which consistently show that TXA reduces bleeding without increasing thromboembolic events [3, 19]. While encouraging, the present study was not powered to detect rare complications. The absence of symptomatic venous thromboembolism within 30 days should therefore be interpreted with caution, and validation in larger multicenter cohorts remains necessary. Nonetheless, the combination of efficacy and reassuring safety renders TXA an attractive adjunct for routine prophylaxis in SG.

### Independent Predictor of Improved Outcomes

Multivariate regression confirmed TXA as an independent predictor of reduced EBL. Its effect remained significant even after adjusting for common clinical and surgical covariates, including BMI, operative time, age, sex, diabetes mellitus, and hypertension. Notably, none of these variables, often implicated in perioperative bleeding risk, showed significant associations in our model.

This strengthens the inference that TXA’s hemostatic benefit is direct rather than confounded by baseline patient characteristics or operative factors. Such statistical independence provides compelling support for its routine incorporation into perioperative care in sleeve gastrectomy, reinforcing the notion that TXA appears to exert a direct hemostatic effect independent of patient or surgical covariates, suggesting it may serve as a useful adjunct to existing perioperative strategies.

TXA’s effect as an independent determinant of lower bleeding is consistent with external evidence. RCTs and controlled studies in SG show fewer bleeding events and staple-line bleeding with TXA, without excess VTE [11, 13]. Meta-analyses in MBS report materially lower postoperative bleeding with TXA and no increase in thromboembolism [3, 6].Large real-world cohorts also demonstrate reduced postoperative bleeding after TXA use, supporting effectiveness beyond trial settings [20]. While few SG trials publish multivariable models, contemporary protocols prespecify adjusted analyses for bleeding endpoints, underscoring that our adjusted finding align with current methodological standards [21].

### Strengths and Limitations

The study has several strengths. The randomized controlled design minimizes selection bias and supports a causal inference between TXA administration and reduced bleeding. The use of multivariate regression further strengthens internal validity by confirming TXA’s independent effect after adjusting for clinical and surgical covariates. In addition, detailed intraoperative and postoperative data collection, coupled with 6 months of follow-up, provided a robust dataset for assessing both immediate and sustained outcomes.

However, some limitations must be acknowledged. Being a single-center study may limit generalizability, and the sample size, while adequate for primary endpoints, is underpowered to detect rare but serious complications such as venous thromboembolism, transfusion, or mortality. Based on meta-analytic estimates of TXA safety in bariatric and orthopedic surgery, detecting even a 1% absolute increase in VTE risk (baseline ≈ 0.3%) would require more than 5,000 participants per arm—well beyond the scope of this study. Additionally, the interpretation of postoperative “melena” warrants clarification. In our dataset, the term was used descriptively based on nursing- or patient-reported dark stools rather than endoscopically confirmed bleeding. None of these cases required transfusion, intervention, or demonstrated hemodynamic instability. The small sample size likely inflated the apparent 1.5–3% rate, as a few minor events can disproportionately affect percentage estimates in limited cohorts.

The observed reduction in postoperative WBC counts raises the hypothesis of an anti-inflammatory effect, but this was not directly investigated. Larger multicenter trials with mechanistic analyses are needed to validate these findings and further explore TXA’s biological effects in metabolic and bariatric surgery. Finally, the absence of staple-line reinforcement and the modest sample size further limit external validity. These factors should be considered when interpreting the findings, and larger multicenter randomized trials are warranted to confirm both efficacy and safety across broader populations.

The absence of staple-line reinforcement represents another limitation of this study. While this ensured procedural uniformity across participants, it may restrict external validity, as centers routinely using buttressing or oversewing might experience different bleeding profiles. Furthermore, meticulous omental and short gastric vessel sealing with LigaSure™ could have independently contributed to reduced bleeding, potentially amplifying the perceived hemostatic benefit of tranexamic acid.

### Future Directions

Recent evidence offers a more nuanced perspective on the role of tranexamic acid in sleeve gastrectomy. Dalkılıç et al. [22] demonstrated that intraoperative TXA combined with omentopexy and oversewing significantly reduced postoperative hemoglobin decline, whereas Lourie et al. [23] observed no difference in bleeding or thromboembolic events following a single preoperative dose. These divergent findings suggest that TXA alone may not be the determining factor in perioperative bleeding control; rather, its efficacy appears to depend on the integration of pharmacologic and technical measures such as vessel sealing, staple-line reinforcement, and timing of administration. Accordingly, TXA should be regarded as an adjunct within a multimodal hemostatic strategy rather than a stand-alone intervention. Nevertheless, its ability to attenuate intraoperative and occult bleeding may still translate into reduced morbidity among patients who experience bleeding-related complications, potentially improving recovery and minimizing transfusion or reoperation risk.

Future research should therefore extend beyond simple efficacy assessments toward larger, methodologically complex randomized controlled trials that stratify patients by surgical technique, TXA timing and dosage, and baseline bleeding risk. Such trials should also integrate mechanistic analyses—evaluating inflammatory, hematologic, and microvascular parameters—to clarify whether TXA exerts additional anti-inflammatory or endothelial-stabilizing effects. Ultimately, defining TXA’s role within a comprehensive, evidence-based hemostatic framework could refine perioperative protocols in metabolic and bariatric surgery and enhance both safety and efficiency of care.

## Conclusion

In this single-center study, tranexamic acid administration during sleeve gastrectomy was associated with reduced perioperative blood loss and hemoglobin decline, without evidence of increased thrombotic or other postoperative complications. These findings suggest that TXA may be a valuable adjunct to standard hemostatic measures, offering potential benefits in improving perioperative outcomes. However, given the study’s sample size, our results should be interpreted with caution. Larger, multicenter randomized trials are needed to confirm the safety and efficacy of TXA across broader patient populations and to establish its role within routine perioperative care for sleeve gastrectomy.

## Data Availability

No datasets were generated or analysed during the current study.
